# Supplementary material for: A Novel Dual PI3K/mTOR Inhibitor, XIN-10, for the Treatment of Cancer
Source: Int J Mol Sci. 2023 Oct 1;24(19):14821. doi: 10.3390/ijms241914821 (PMC10573424; doi:10.3390/ijms241914821)
Supplement: Supplementary file 1 [file ijms-24-14821-s001.zip › ijms-2566376-supplementary.pdf]

Supplementary Material

A novel dual PI3K/mTOR inhibitor, XIN-10, for the treatment of breast cancer

Leixuan Luo †, Xin Sun †, Yang Yang, Lulu Xia, Shiyu Wang, Yuxing Fu, Yuxuan Zhu, Shan Xu * and Wufu Zhu *

Jiangxi Provincial Key Laboratory of Drug Design and Evaluation, School of Pharmacy, Jiangxi Science & Technology Normal University, 605 Fenglin Road, Nanchang 330013, China; luoleixuan8@163.com (L.L.); sunxin_haha@163.com (X.S.); yangyang9879876@163.com (Y.Y.); 17839837110@163.com (L.X.); wangsy030101@163.com (S.W.); fformd@163.com (Y.F.); zyuxuan1998@163.com (Y.Z.)

* Correspondence: xush@jxstnu.edu.cn (S.X.); zhuwf@jxstnu.edu.cn (W.Z.)

† These authors contributed equally to this work.

Table of Contents

Figure S1. Synthesis of XIN-10

Figure S2. ^{1}H NMR Spectral of XIN-10

Figure S3. ^{13}C NMR Spectral of XIN-10

Figure S4. TOF MS analytical data of XIN-10

Figure S5. LCMS traces of XIN-10

Figure S1. Synthesis of XIN-10

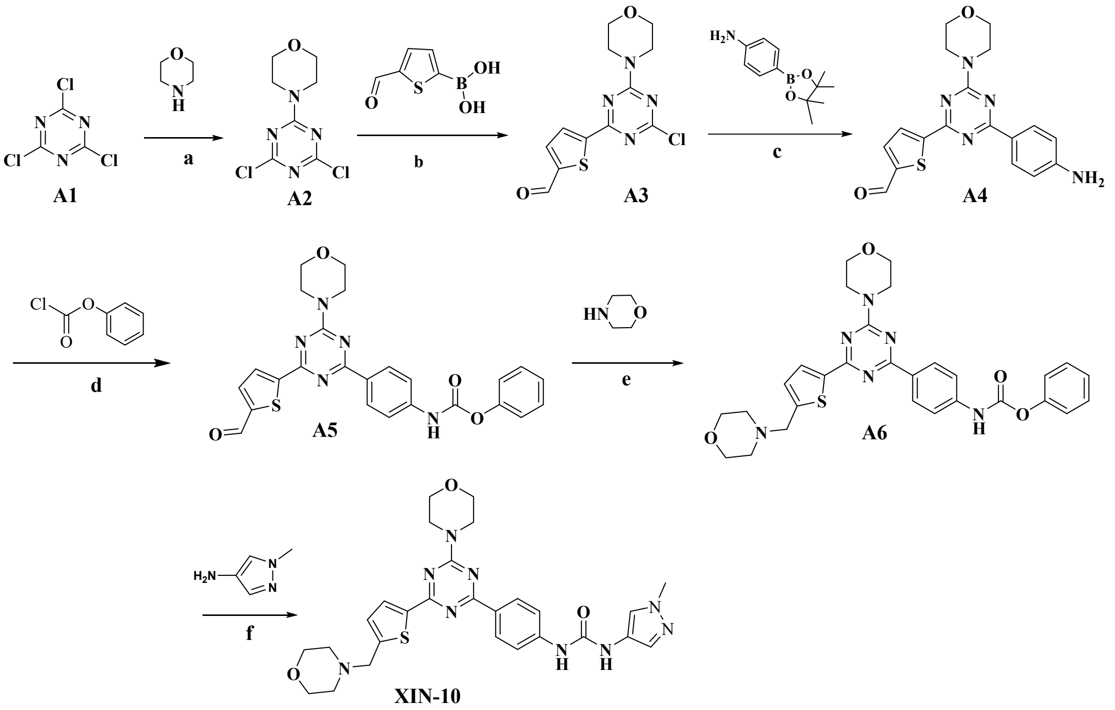

Figure S1: (a) Morpholine, Methylene chloride, 0 ºC, 2 h; (b) Ethylene glycol dimethyl ether, H2O, K2CO3, Pd(PPh3)Cl2, 75 ºC, 24 h; (c) Ethylene glycol dimethyl ether, H2O, K2CO3, Pd(PPh3)Cl2, 125 ºC, 6 h; (d) Phenyl carbonochloridate, DMAP, r.t, 2 h. (e) $(CH_3COO)_3BHNa$, 1,2-Dichloroethane, Acetic acid, r.t., 48 h; (f) Acetonitrile, DIPEA, 55 ºC~85 ºC, 6 h.

Figure S2. ^{1}H NMR Spectral of XIN-10

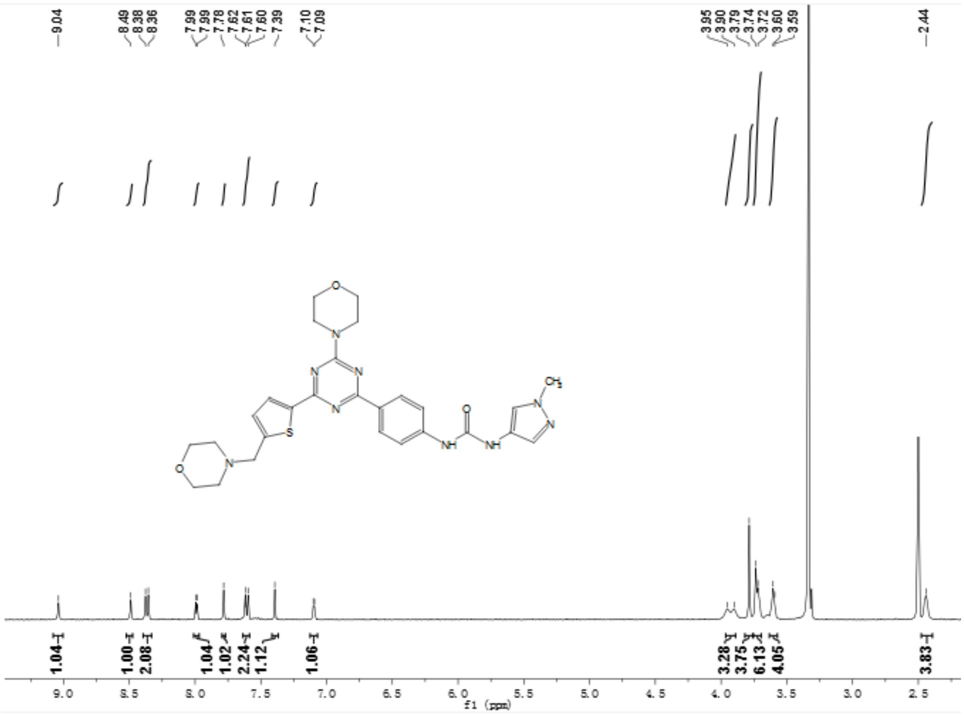

Figure S3. ^{13}C NMR Spectral of XIN-10

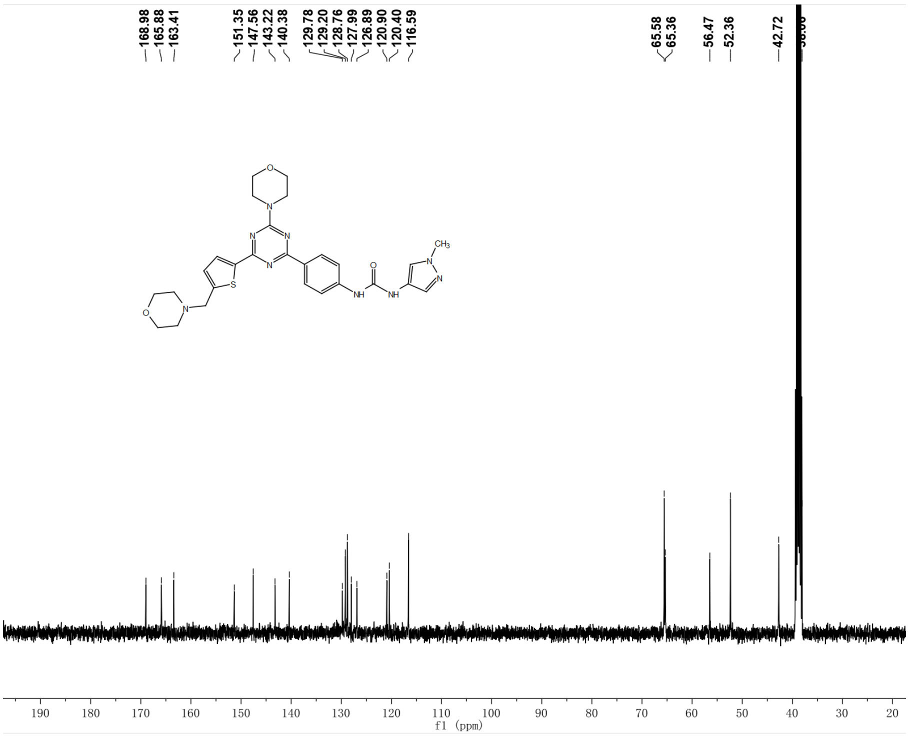

Figure S4. TOF MS analytical data of XIN-10

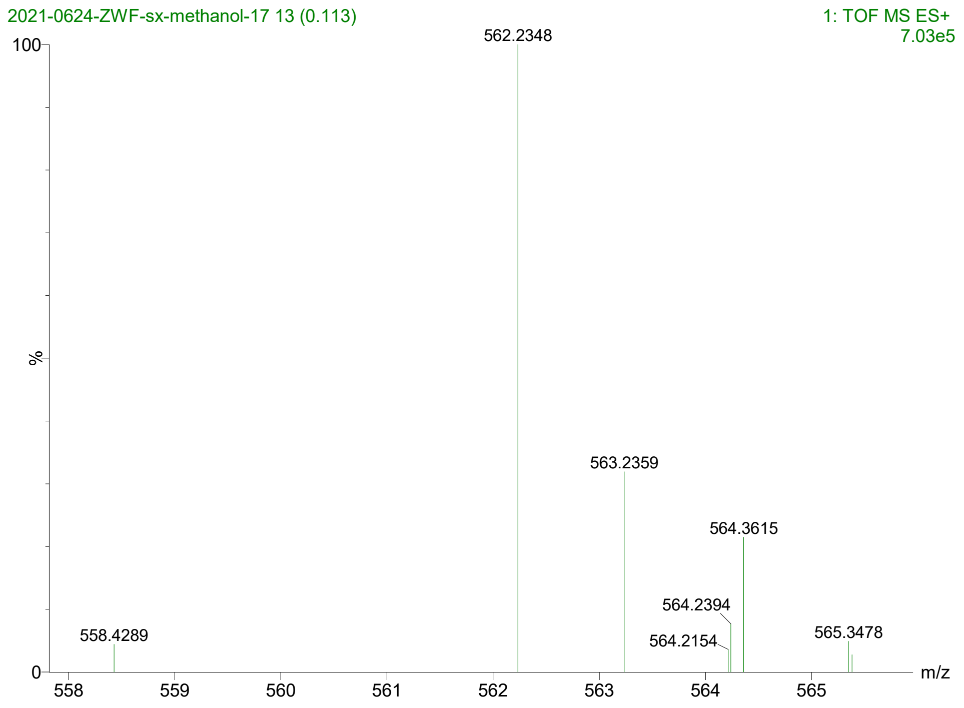

Figure S5. LCMS traces of XIN-10

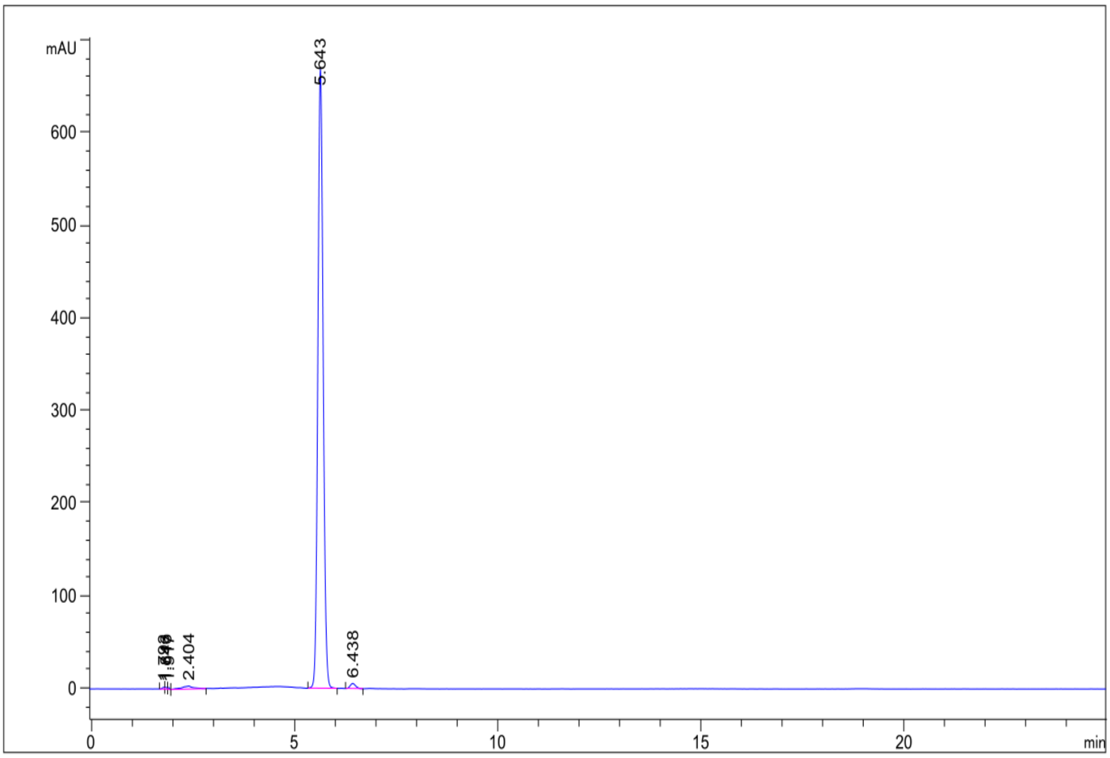


```
=======================================================================
                         Area Percent Report
=======================================================================

Sorted By                      :      Signal
Multiplier                     :      1.0000
Dilution                       :      1.0000
Use Muitiplier & Dilution Factor With ISTDs

Signal 1: DAD A, Sig=310,16
```

peak #	RetTime [min]	Type	Width [min]	Area [mAU*s]	Height [mAU]	Area %
1	1.793	BV	0.0702	8.88499	1.98485	0.1443
2	1.846	VV	0.0480	7.14517	2.08313	0.1161
3	1.917	VV	0.0463	5.69701	1.93952	0.0926
4	2.404	VB	0.2684	66.35707	3.16783	1.0780
5	5.643	BB	0.1390	6015.51807	668.43378	97.7285
6	6.438	BB	0.1483	51.73173	5.47123	0.8404
Totals :				6155.33404	683.08032	

```
=======================================================================
                        *** End of Report ***
```
